# Differences in the Microbial Composition of Hemodialysis Patients Treated with and without β-Blockers

**DOI:** 10.3390/jpm11030198

**Published:** 2021-03-12

**Authors:** Yi-Ting Lin, Ting-Yun Lin, Szu-Chun Hung, Po-Yu Liu, Wei-Chun Hung, Wei-Chung Tsai, Yi-Chun Tsai, Rachel Ann Delicano, Yun-Shiuan Chuang, Mei-Chuan Kuo, Yi-Wen Chiu, Ping-Hsun Wu

**Affiliations:** 1 Department of Family Medicine, Kaohsiung Medical University Hospital, Kaohsiung 80708, Taiwan; 960254@kmuh.org.tw (Y.-T.L.); kinkipag@gmail.com (Y.-S.C.); 2 Faculty of Medicine, College of Medicine, Kaohsiung Medical University, Kaohsiung 80708, Taiwan; lidam65@yahoo.com.tw (Y.-C.T.); mechku@kmu.edu.tw (M.-C.K.); chiuyiwen@kmu.edu.tw (Y.-W.C.); 3 Graduate Institute of Clinical Medicine, College of Medicine, Kaohsiung Medical University, Kaohsiung 80708, Taiwan; 4 Division of Nephrology, Taipei Tzu Chi Hospital, Buddhist Tzu Chi Medical Foundation, New Taipei City 23142, Taiwan; water_h2o_6@hotmail.com (T.-Y.L.); szuchun.hung@gmail.com (S.-C.H.); 5 School of Medicine, Tzu Chi University, Hualien 97071, Taiwan; 6 Department of Internal Medicine, College of Medicine, National Taiwan University, Taipei 100225, Taiwan; poyu.liu@gmail.com; 7 Department of Microbiology and Immunology, Kaohsiung Medical University, Kaohsiung 80708, Taiwan; wchung@kmu.edu.tw; 8 Division of Cardiology, Department of Internal Medicine, Kaohsiung Medical University Hospital, Kaohsiung Medical University, Kaohsiung 80708, Taiwan; k920265@gap.kmu.edu.tw; 9 Division of General Medicine, Kaohsiung Medical University Hospital, Kaohsiung Medical University, Kaohsiung 80708, Taiwan; 10 Faculty of Renal Care, College of Medicine, Kaohsiung Medical University, Kaohsiung 80708, Taiwan; 11 Institute of Surgical Sciences, Uppsala University, 752 36 Uppsala, Sweden; rachel.delicano@surgsci.uu.se; 12 Division of Nephrology, Department of Internal Medicine, Kaohsiung Medical University Hospital, Kaohsiung Medical University, Kaohsiung 80708, Taiwan

**Keywords:** microbiome, beta-blocker, hemodialysis, next-generation sequencing, propensity score matching methods

## Abstract

β-blockers are commonly prescribed to treat cardiovascular disease in hemodialysis patients. Beyond the pharmacological effects, β-blockers have potential impacts on gut microbiota, but no study has investigated the effect in hemodialysis patients. Hence, we aim to investigate the gut microbiota composition difference between β-blocker users and nonusers in hemodialysis patients. Fecal samples collected from hemodialysis patients (83 β-blocker users and 110 nonusers) were determined by 16S ribosomal RNA amplification sequencing. Propensity score (PS) matching was performed to control confounders. The microbial composition differences were analyzed by the linear discriminant analysis effect size, random forest, and zero-inflated Gaussian fit model. The α-diversity (Simpson index) was greater in β-blocker users with a distinct β-diversity (Bray–Curtis Index) compared to nonusers in both full and PS-matched cohorts. There was a significant enrichment in the genus *Flavonifractor* in β-blocker users compared to nonusers in full and PS-matched cohorts. A similar finding was demonstrated in random forest analysis. In conclusion, hemodialysis patients using β-blockers had a different gut microbiota composition compared to nonusers. In particular, the *Flavonifractor* genus was increased with β-blocker treatment. Our findings highlight the impact of β-blockers on the gut microbiota in hemodialysis patients.

## 1. Introduction

The gut microbiota has a crucial role in metabolic, nutritional, physiological, defensive, and immunological processes in the human body, with its composition linked to human health and the development of diseases [1,2]. Human-microbiome association can be considered as integration in evolution. The microbiome can modulate and restore human health [3]. Changes in this microbial equilibrium, that is, dysbiosis, promotes and influences the course of many intestinal and extra-intestinal diseases [4,5,6]. In addition to genetic and environmental factors, several common medications (e.g., proton pump inhibitors, nonsteroidal anti-inflammatory drugs, atypical antipsychotics, selective serotonin reuptake inhibitors, antibiotics, statins, and antidiabetic drugs) are associated with the specific gut microbiota composition [7,8,9,10,11,12,13]. Indeed, drug-microbiome-host interactions are complex and multifactorial, impacting host metabolism [14,15]. Hence, they should be part of the core phenotype set for human gut microbiota research [16].

Patients with chronic kidney disease (CKD) have altered gut microbiota, with a bidirectional causal effect relationship [17,18]. Among the cardiovascular preventive drugs for patients with end-stage renal disease (ESRD), β-blockers are commonly prescribed in higher cardiovascular risk patients to prevent sudden cardiac death [19,20]. Beyond the clinical effect of β-blockers in ESRD patients, they also have a potential impact on gut microbiota [7,16]. Besides, the benefit of beta-blockers may be attributed to preventing the activity of the gut microbe-generated metabolite, such as phenylacetylglutamine [21]. However, limited study has investigated the impact on ESRD patients. Herein, we evaluate the gut microbiota composition of β-blocker users and nonusers in Taiwanese hemodialysis patients.

## 2. Materials and Methods

### 2.1. Study Participants

The Ethics Committee approved the study protocols of Kaohsiung Medical University Hospital (KMUHIRB-E(I)-20160095 and KMUHIRB-E(I)-20180118) and Taipei Tzu Chi Hospital (07-X01-002). All participants provided written informed consent. Hemodialysis patients were recruited from the dialysis unit of Taipei Tzu Chi Hospital and Kaohsiung Medical University Hospital in Taiwan from August 2017 to February 2018. The inclusion criteria were patients with age more than 18 years old and received regular hemodialysis three times per week, 3.5–4 h with high-flux dialyzers. The exclusion criteria included patients with partial or total colectomy, inflammatory bowel diseases, active malignancies, or patients who were prescribed antibiotics within three months before enrollment. Fecal samples were collected from 193 stable hemodialysis patients and analyzed by high-throughput 16S ribosomal RNA gene sequencing to compared participants with and without β-blocker treatment. All β-blocker users were prescribed for at least one month.

### 2.2. Comorbidity, Laboratory, and Clinical Variables

All baseline characteristics of sociodemographic data, age, sex, body mass index, dialysis vintage, arteriovenous shunt type, comorbidities, medications, and biochemical data were collected in the built-in electronic health care system. Blood samples were collected after overnight fasting through the arteriovenous fistula or graft before scheduled hemodialysis sessions. The biochemical data included serum values for hemoglobin, albumin, high sensitivity C reactive protein, total cholesterol, low-density lipoprotein, triglycerides, ion calcium, and phosphate from routine blood samples obtained within 30 days before stool sample collection. Diet was evaluated by a licensed dietitian using a modified short-form food frequency questionnaire. No specific antioxidant supplements (i.e., tea, cocoa products, or wine) were recorded because of strict dietary restrictions in hemodialysis patients. Participants have followed the nutrition guideline of the National Kidney Foundation’s Kidney Disease Outcomes Quality Initiative (KDOQI™) [22], which recommends a high-protein intake (1.1–1.4 g/kg/day) and reduced consumption of fruits, vegetables, and dietary fiber to avoid potassium overload. Diabetes was defined as HbA1C 6.5% or higher or use of oral antidiabetic agents or insulin. Hypertension was defined as 140/90 mmHg or higher or taking blood pressure-lowering drugs. A history of myocardial infarction or documented by coronary angiography, class III or IV congestive heart failure, or a cerebrovascular accident were defined as cardiovascular disease.

### 2.3. Fecal Sample Collection and Bacterial 16S rRNA Amplicon Sequencing and Processing

All stool samples were frozen immediately after collection by each participant, then delivered in cooler bags to the laboratory (Germark Biotechnology, Taichung, Taiwan) within 24 h. A QIAamp DNA Stool Mini Kit (Qiagen, MD, USA) was used to extract DNA from fecal samples. Barcode-indexed PCR primers (341F and 805R) were used to create an amplicon library by amplifying the variable regions 3 and 4 (V3–V4) of the 16S rRNA gene [23]. The amplicons were sequenced (300 bp paired-end) using an Illumina MiSeq sequencer at the same time in the same laboratory to avoid batch effects (Germark Biotechnology, Taichung, Taiwan). The 16S-amplicon pipeline was adapted from 16S Bacteria/Archaea SOP v1 of Microbiome Helper workflows [24]. Paired-End reAd mergeR (PEAR; version 0.9.8) [25] was used to merge paired-end reads to raw reads, then filtered low-quality reads by thresholds of sequence length ≥400 bp and quality score of 90% bases of reads ≥20. Quantitative Insight Into Microbial Ecology (QIIME; version 1.9.1) software was used to select operational taxonomic units (OTU) [26]. The SILVA (version 123) 16S database [27,28] was applied to cluster OTUs and assign taxonomy using the UCLUST algorithm (version v1.2.22q) [29] with a 97% sequence identity threshold. Reads were dereplicated, and singletons were discarded. The final OTU table was rarefied into minimum sequencing depth in the data set.

### 2.4. Propensity Score Matching

**Figure 1:**
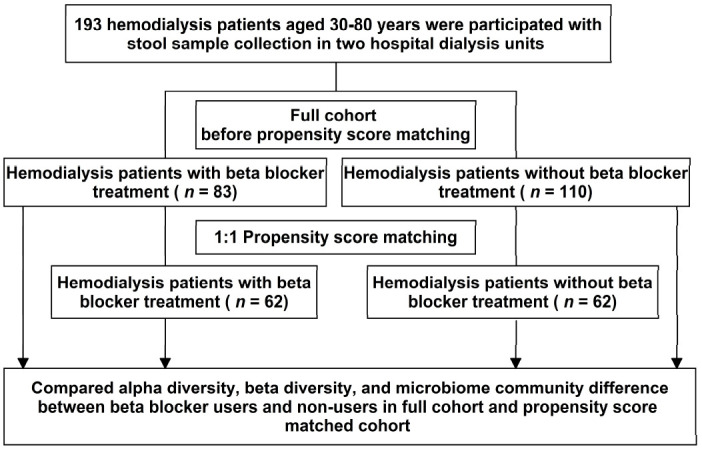
Study design.

Propensity score (PS) matching [30,31] was performed to balance confounders between the comparisons of interest (i.e., β-blocker users versus nonusers) and minimize the confounding by indication resulting from nonrandom treatment study. Using a logistic regression model, β-blocker use was accessed to estimate the propensity to receive a β-blocker for each participant based on potential confounders, including age, sex, body mass index, dialysis vintage, smoking history, vascular access type, Bristol stool scale, dietary intake, comorbidities (diabetes mellitus, hypertension, dyslipidemia, coronary artery disease, heart failure, cerebrovascular disease, and parathyroidectomy history), concomitant drugs used (including ACEI (angiotensin converting enzyme inhibitors)/ARB (angiotensin-receptor blockers), glucose-lowering drugs (such as sulfonylurea, dipeptidyl peptidase-4 inhibitors, insulin), statin, calcium carbonate, and proton pump inhibitors), and clinical laboratory data (hemoglobin, albumin, total cholesterol, triglyceride, high sensitivity C reactive protein (hsCRP), sodium, potassium, total calcium, phosphate, parathyroid hormone, serum iron, ferritin, normalized protein catabolic rate (nPCR), and single pool Kt/V). In this study, 193 hemodialysis patients were enrolled, including 83 β-blocker users and 110 nonusers (full cohort). PS-matched (1:1) analysis was used to match participants with β-blocker treatment (*N* = 62) to participants without β-blocker treatment (*N* = 62) (PS-matched cohort, Figure 1).

### 2.5. Statistical and Bioinformatics Analyses of Microbiota

The study design is presented in Figure 1. Demographic characteristics are shown as the mean, median, or frequency, with differences between β-blocker users and nonusers determined using an independent T-test or chi-squared test, as appropriate. A rarefaction curve was built to prevent methodological artifacts originating from variations in sequencing depth. The α-diversity indices (Shannon and Simpson’s indices) estimated the evenness of taxa within each sample and were generated using the R “vegan” package and calculated the *p*-value by the Kruskal–Wallis test. The β-diversity provides a comparison of the taxonomic profiles’ differences between pairs of individual samples. The β-diversity was calculated based on the Bray–Curtis distance matrices and was visualized using principal coordinates analysis (PCoA) and calculated using homogeneity of group dispersions by Permutational Analysis of Multivariate Dispersions (PERMDISP) [32].

Co-occurrence analysis was used to determine the relationships within communities, with core microbiome analysis performed at the genus level using MicrobiomeAnalyst [33], in which sample prevalence and relative abundance cut-off values were set at 20 and 0.2%, respectively. For visualization of the internal interactions and further measurement of the microbial community, Sparse Correlations for Compositional data (SparCC) was used to calculate the Spearman correlation coefficient with the corresponding *p*-value between every two taxa. Microbiota community structure was assessed by co-occurrence networks built by the SparCC algorithm [34]. The *p*-values were estimated by 100 random permutations and iterations for each SparCC calculation, and correlation matrices were computed from the resampled data matrices. Only OTUs with correlation scores greater than 0.4 and *p*-value less than 0.05 were categorized into co-abundance groups (CAGs); these coefficients were also used to assess the length of edges on the network. An undirected network, weighted by SparCC correlation magnitude, was generated using bioinformatics tools in MicrobiomeAnalyst [33].

The bacterial OTU difference between β-blocker users and nonusers was analyzed by the linear discriminant analysis (LDA) of effect size (LEfSe) analysis with samples presenting more than 0.1% relative abundance and found >30% of all samples. The LEfSe analysis employed the nonparametric factorial Kruskal–Wallis test or Wilcoxon rank-sum test and LDA to identify differentially abundant taxa between the groups. Only taxa with LDA score greater than two or less than two at a *p*-value < 0.05 were considered significantly enriched. All statistical tests are two-tailed, and a *p*-value < 0.05 was considered statistically significant. The random forest method [35] was performed to determine a ranked list of all bacterial taxa to identify the most predictive bacterial community to classify β-blocker users and nonusers. The random forest is a supervised learning algorithm ranking OTUs based on their ability to discriminate among the groups, while accounting for the complex interrelationships in high dimensional data. The MetagenomeSeq method was also used to evaluate differential abundance in sparse marker-gene survey data using a zero-inflated Gaussian (ZIG) fit model to account for undersampling and sparsity in OTU count data after normalizing the data through cumulative sum scaling (CSS) [36]. Finally, the log-transformed read counts difference of the top selected genera from the ZIG fit model between β-blocker users and nonusers was analyzed in the full and PS-matched cohorts.

Co-occurrence and random forest analyses were performed by MicrobiomeAnalyst [33]. The other statistical analyses were performed using R statistical software (version 3.5.1) and STATA statistical software (version 14; StataCorp LLC, College Station, TX, USA).

### 2.6. Functional Prediction Analysis

Phylogenetic Investigation of Communities by Reconstruction of Unobserved States (PICRUSt2) [37] was used to predict the metagenome, which was based on Integrated Microbial Genomes (IMG) database [38] to evaluate the functions of gut microbiota among β blocker users and nonusers in the full cohort and PS-matched cohort. An OTU table was used for predicting metagenome based on Kyoto Encyclopedia of Genes and Genomes (KEGG) Orthology (KO) annotations. Metabolic module enrichment analysis was done with functional sets enrichment analysis (FSEA) described by Liu et al. [39]. The ‘FSEA’ function in the MARco R package based on the Liu et al. paper was applied in this study [39]. The ‘FSEA’ was embedded with the ‘gage’ R-package [40]. Enrichment scores were scored based on the GSEA algorithm of the Database for Annotation, Visualization, and Integrated Discovery (DAVID) bioinformatics resources [41,42].

## 3. Results

### 3.1. Patient Characteristics

Patient characteristics are shown in Table 1, with those receiving β-blockers having a higher proportion of diabetes, hypertension, dyslipidemia, coronary artery disease, heart failure, cerebrovascular disease, and more commonly used ACEI/ARB, glucose-lowering drugs (such as dipeptidyl peptidase-4 inhibitors or insulin) and statin. PS matching resulted in 62 matched pairs with balanced baseline characteristics (Table 1).

### 3.2. Gut Microbiota Profile Differs in Hemodialysis Patients with and without β Blocker Treatment

**Figure 2:**
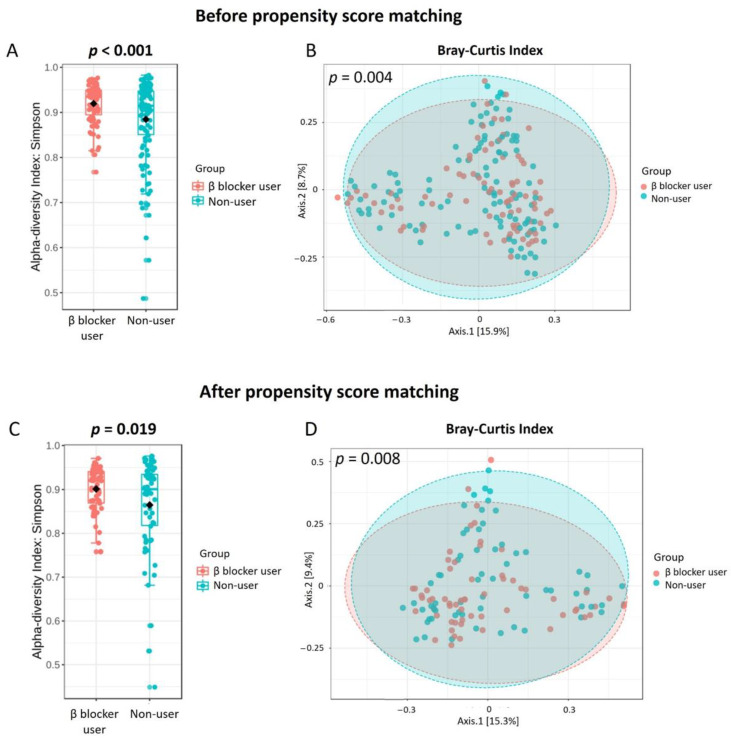
The α-diversity and β-diversity in hemodialysis patients with and without β blocker used in full cohort (**A**,**B**) and propensity score matching cohort (**C**,**D**). β blocker users had a higher α-diversity than β blocker nonusers in full cohort (**A**) and propensity score matching cohort (**C**) β blocker users had a different β-diversity (Bray–Curtis index) compared to β blocker nonusers in full cohort (**B**) and propensity score matching cohort (**D**). The β-diversity *p*-value was calculated using the homogeneity of group dispersions by the Permutational Analysis of Multivariate Dispersions (PERMDISP) test.

The rarefaction curves were close to asymptotic based on the number of OTUs observed. To represent the microbiome community with enough coverage, the rarefaction curves reached saturation at a cutoff point of 45,000 sequences per sample (Supplementary Figure S1). Compared to the gut microbiota composition and structure between β-blocker users and nonusers, no substantial differences were observed in the relative abundance proportion in the full and PS-matched cohorts (Supplementary Figure S2). Hemodialysis patients taking β-blockers had a higher α-diversity and a distinct β-diversity compared to nonusers in the full and PS-matched cohorts (Figure 2). The core microbiome was Bacteroides in hemodialysis patients (Supplementary Figure S3A), with a similar core microbiome in β-blocker users and nonusers (Supplementary Figure S3B).

### 3.3. Specific Microbial Taxa Differences between β-Blocker Users and Nonusers

**Figure 3:**
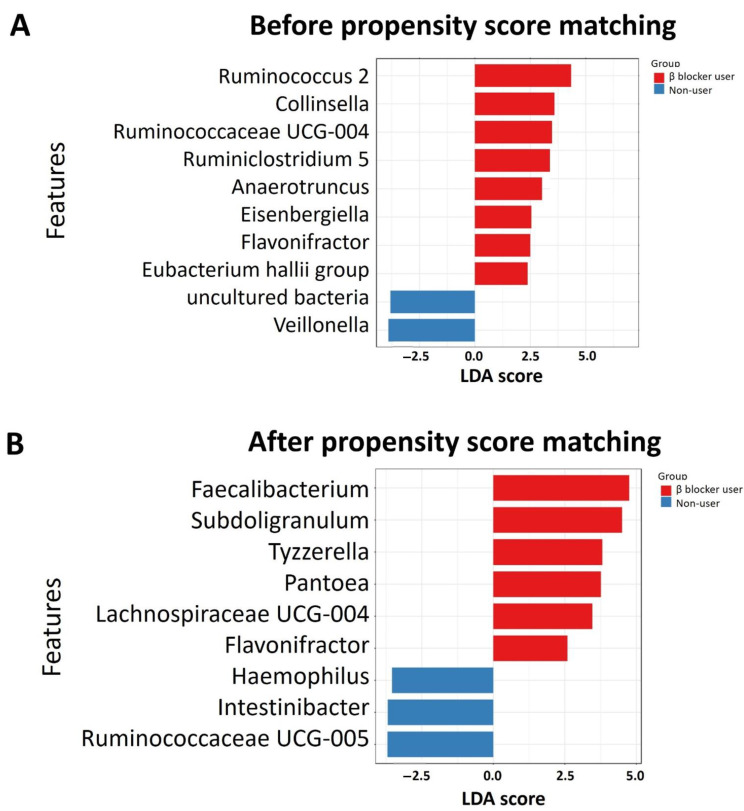
Taxonomic differences were detected between β blocker users and nonusers in the full cohort (**A**) and propensity score matching cohort (**B**). Linear discriminative analysis (LDA) effect size (LEfSe) analysis between β blocker users (red) and nonusers (blue) with an LDA score > 2.0 or < −2 with *p*-value > 0.1 among β blocker users and nonusers.

**Figure 4:**
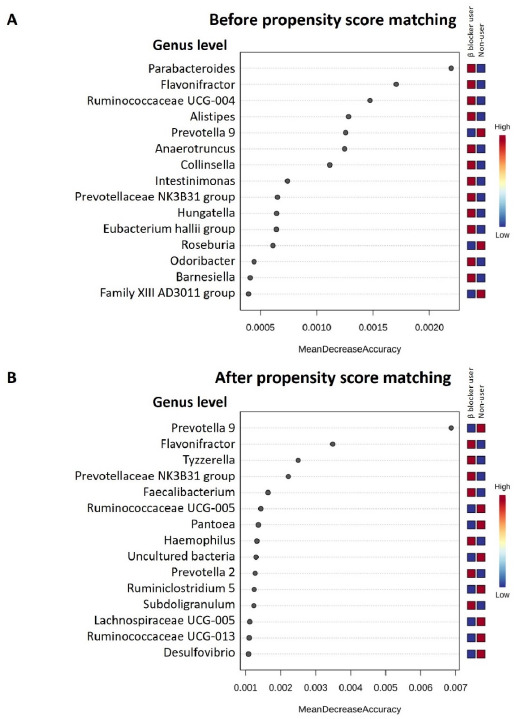
Determination of specific bacteria for discriminatory across hemodialysis patients with and without β blocker treatment in full cohort (**A**) and propensity score matching cohort (**B**). The discriminatory taxa were determined by applying Random Forest analysis using the genus-level abundance.

Discriminant analysis using LEfSe identified the significant differentiating taxa between study groups. In the full cohort, the genera *Ruminococcus 2, Collinsella, Ruminococcaceae UCG-004, Ruminiclostridium 5, Anaerotruncus, Eisenbergiella,* and *Flavonifractor* were enriched in β-blocker users compared to nonusers (Figure 3A). In the PS-matched cohort, the enriched genera were *Faecalibacterium, Subdoligranulum, Tyzzerella, Pantoea, Lachnospiraceae UCG-004,* and *Flavonifractor* were found (Figure 3B). Using random forest models for taxonomy prediction, the top three ranked genera to discriminate between β-blocker users and nonusers were *Parabacteroides, Flavonifractor,* and *Ruminococcaceae UCG-004* in the full cohort (Figure 4A), *Prevotella 9, Flavonifractor,* and *Tyzzerella* in the PS-matched cohort (Figure 4B).

**Figure 5:**
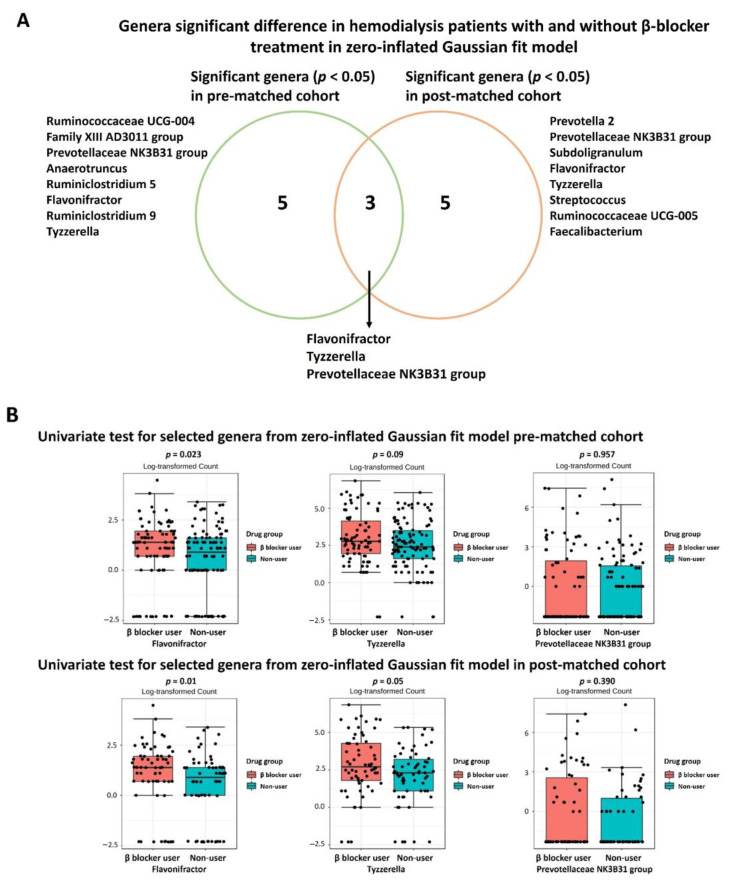
The genera difference between β blocker users and nonusers in the full cohort and propensity score matching cohort using zero-inflated Gaussian fit model. (**A**) The Venn diagram showed the different significant genera in the full cohort and propensity score-matched cohort. (**B**) Univariate test between selected genera from zero-inflated Gaussian fit model. Significance was considered for *p* < 0.05.

To reduce the effect of zero-inflation in the microbiome data, we performed the MetagenomeSeq algorithm integrating the CSS method and a statistical model based on the ZIG distribution. Evaluating the significant difference in genus taxonomy between β-blocker users and nonusers, we found eight genera differences in the full cohort and PS-matched cohort (Supplementary Table S1). There were three different genera (*Flavonifractor, Tyzzerella,* and *Prevotellaceae NK3B31 group*) in both the full and PS-matched cohorts (Figure 5A). Focusing on the ZIG fit model to predict specific genera, there was an increased *Flavonifractor* genus in β-blocker users compared to nonusers using a classical univariate test (Kruskal–Wallis test) in the full (*p* = 0.023) and PS-matched cohorts (*p* = 0.01) (Figure 5B). However, no differences were found in *Tyzzerella* or *Prevotellaceae NK3B31 group* (Figure 5B).

Using PICRUSt2 as a metagenome predictive exploratory tool, genes were categorized into KEGG Orthology metabolic pathways. All predicted KEGG Orthology (KOs) were mapped to KEGG metabolic pathways. Each pathway was tested with gene-set enrichment by comparing expected gene abundance between β blocker users and nonusers in full and PS-matched cohorts. However, no significant KEGG enriched pathways were observed (Figures S4 and S5).

## 4. Discussion

In the present study, hemodialysis patients treated with β-blockers had a higher α-diversity and a distinct β-diversity compared to nonusers. The microbial communities contained higher levels of *Bacteroidetes* and lower levels of *Firmicutes* in all hemodialysis patients, which is similar to CKD rat microbial communities [43] and in a human CKD microbiota study [44]. Co-occurrence analysis revealed no difference in keystone taxa *Bacteroides* between β-blocker users and nonusers. Overall, there was an enriched genus *Flavonifractor* in β-blocker users in the full and PS-matched cohorts. Furthermore, LEfSe analysis, random forest algorithm, ZIG fit model, and univariate test all confirmed this difference between groups. However, we did not determine KEGG metabolic pathways between β blocker users and nonusers using PICRUSt2 functional prediction analysis.

β-blocker use was associated with a higher α-diversity than nonusers in hemodialysis patients, which was linked to a favorable healthy state [45]. Increased α-diversity has been associated with foods generally considered healthy, such as plant consumption or red wine [46,47,48]. Furthermore, commonly used medications such as antibiotics or proton pump inhibitors can decrease gut α-diversity [49]. Regarding the specific taxonomy of the gut microbiome, the genus *Flavonifractor* was enriched in β-blocker users in both the full and PS-matched cohorts. *Flavonifractor* is associated with several diseases, such as obesity [50], atrial fibrillation [51], coronary artery disease [52], and medications (antidiabetic drugs, such as Metformin and Glucagon-like peptide 1 Receptor agonist [53]). It can convert quercetin or other flavonoids into acetic acid and butyric acid [54] and is also correlated with oxidative stress and inflammation [55]. The presence of *Flavonifractor* was found in association with circulating inflammatory markers (i.e., interleukin-6, interleukin-8, interleukin-1β) [56], which were linked to cardiovascular disease. Besides, oral administration of *Flavonifractor plautii* was involved in the inhibition of tumor necrosis factor-α expression in obese adipose tissue inflammatory environments [57]. Thus, the increased abundance of *Flavonifractor* by β-blocker treatment may have a potential benefit in cardiovascular disease via gut microbiota regulation.

We also identified a potential link between β-blocker use and the genus *Tyzzerella* in the PS-matched cohort. Importantly, *Tyzzerella* was enriched in those with a high cardiovascular risk profile [58]. However, the small sample size limited the potential association between β-blocker use and *Tyzzerella* in univariate analysis, so more extensive studies are needed to confirm this association. Regarding the link between β-blocker and microbiota changes; a chimera mouse model suggested bone marrow beta1/2 adrenergic receptor signaling can regulate host-microbiota interactions, leading to the generation of novel anti-inflammatory treatments for gut dysbiosis [59]. Therefore, depletion of this sympathetic regulation in bone marrow promotes beneficial shifts in gut microbiota associated with gut immune suppression [59]. It is proposed that beta-blockers may provide a beneficial microbiome in such conditions.

We compared the microbiota differences between β-blocker users and nonusers using PS matching analysis in the present study. Since β-blocker intake is highly correlated with age, cardiovascular risk, comorbidities, and concurrent medication, each factor represents a relevant confounder for microbiome analyses [16,60]. Most observational studies have controlled for possible confounding variables, but even rigorous data adjustment cannot eliminate the risk of bias. PS matching is an alternative to reduce the effect of influencing factors on gut microbiota analysis [30,31]; thus, we selected variables of interest as potential confounders and then performed PS matching to reduce these effects deviations and confounding variables to conduct a reasonable comparison between groups. The intestinal microbiota was affected by various factors, including demographic data, comorbidities, concomitant medications, and clinical laboratory data, and the application of PS matching eliminated confounding factors. Using PS analysis, there was still a higher α-diversity and different β-diversity in β-blocker users compared to nonusers. We also identified six genera associated explicitly with the β-blocker user in the LEfSe analysis, four top-ranked genera in random forest analysis, and eight genera in ZIG fit model analysis. Although there were some differences in bacterial associations with β-blocker use in our full (before PS matching) and PS-matched cohorts, we investigated the taxa represented in both the full and PS-matched cohorts. Importantly, three genera (*Flavonifractor, Tyzzerella,* and *Prevotellaceae NK3B31 group*) were both significant differences in ZIG fit model among the full and PS-matched cohorts. The genera abundance differences between β-blocker users and nonusers were changed in the PS matching procedure. The genera abundance significant differences in *Ruminiclostridium 9*, *Ruminococcaceae UCG-004*, *Anaerotruncus*, and *Ruminiclostridium 5* were attenuated after PS matching, suggesting that these genera abundances may be more strongly associated with other confounding variables, such as comorbidities or concomitant medications, which was accounted for in the PS models. In specific, genus *Anaerotruncus* was reported related to hypertension [61,62,63], diabetes mellitus [64], and dipeptidyl peptidase 4 inhibitors used [65], which were unbalance in the pre-matched cohort. The genus *Ruminiclostridium* and *Ruminococcaceae* were correlated to hypertension in the previous study [66,67,68]. Thus, the change of gut microbiome difference in β-blockers users and nonusers before and after PS matching reflected confounders’ influence. Since many factors influencing gut microbiota, we performed PS matching as an alternative technique to account for multiple confounders in this study.

In addition, there were more zeros than expected under the assumption of Poisson or negative binomial distributions for microbiome OTU counts, known as zero-inflation. One popular strategy to circumvent the zero-inflation problem is to add a pseudo-count [69]; however, this assumption may not be appropriate due to the large extent of structural zeros due to physical absence. Moreover, the pseudo-count choice is arbitrary, and the clustering results can be highly dependent upon the choice [70]. Thus, CSS was developed for microbiome sequencing data, and a zero-inflated model was used to model read counts that have an excess of zeros [36,71,72]. In CSS, raw counts are divided by the cumulative sum of counts up to a percentile determined using a data-driven approach to capture the relatively invariant count distribution for a dataset. To solve the zero-inflation issue, we applied the ZIG fit model and calculated the CSS. Interestingly, the four genera in the full (*Ruminococcaceae UCG-004*, *Ruminiclostridium 5*, *Anaerotruncus*, and *Flavonifractor*) and PS-matched cohorts (*Flavonifractor*, *Tyzzerella*, *Faecalibacterium*, *Subdoligranulum*) overlapped in the LEfSe analysis and ZIG fit model analysis.

Several limitations should be mentioned. First, cross-sectional studies only provide an impression of the relative abundance of bacterial taxa at a single time point, so causal inference cannot be addressed. Besides, the observational study only demonstrates the association rather than the causality. Second, the microbiota was assessed with a fecal sample, which may differ from microbiota from other parts of the intestine. Besides, 16S rRNA sequencing is limited as it cannot differentiate viable from non-viable bacteria. A significant portion of the taxa identified by sequencing may not be metabolically active. Thus, further study is needed to investigate various samples, such as small intestine or colon mucosal bacteria. Third, PS matching might not fully balance the overall effects of medications or disease severity, such as the dose of medications or the status between controlled and uncontrolled DM. Finally, the study was performed in Asia hemodialysis patients whose diet is different from other populations, so dietary effects on the gut microbiome should be interpreted with caution.

## 5. Conclusions

This study demonstrated that the composition of the gut microbiota was different in hemodialysis patients treated with β-blockers, with a higher level of α-diversity and genus *Flavonifractor*. These findings support the additional benefits of β-blocker treatment, which may mediate the microbiota in hemodialysis patients. However, the functional relevance of the β-blocker induced microbial differences is unclear. Hence, larger prospective treatment naïve studies are warranted to understand the impact of β-blockers on the gut microbiome of CKD patients and their implications for health and disease.

## Figures and Tables

**Table 1:** Baseline characteristics of hemodialysis patients with and without β blocker treatment.

Baseline Characteristics	Before Propensity Score Matching			After Propensity Score Matching		
	β-Blocker Users (*N* = 83)	β-Blocker Nonusers (*N* = 110)	*p*-Value	β-Blocker Users (*N* = 62)	β-Blocker Nonusers (*N* = 62)	*p*-Value
Age (years)	64.3 ± 11.4	65.4 ± 11.2	0.511	64.7 ± 11.6	66.3 ± 11.8	0.446
Male	49 (59.0%)	57 (51.8%)	0.318	37 (59.7%)	28 (45.2%	0.106
Body mass index	23.4 ± 3.25	23.6 ± 3.91	0.708	23.5 ± 3.34	23.5 ± 3.93	0.988
Dialysis vintage (months)	86.24 ± 56.53	96.54 ± 63.21	0.243	93.22 ± 57.61	85.4 ± 55.67	0.444
Smoking history	15 (18.1%)	12 (10.9%)	0.156	9 (14.5%)	6 (9.7%)	0.409
Arteriovenous fistula	75 (90.4%)	99 (90.0%)	0.934	57 (91.9%)	57 (91.9%)	>0.999
Comorbidities						
Diabetes mellitus	45 (54.2%)	34 (30.9%)	0.001	24 (38.7%)	30 (48.4%)	0.277
Hypertension	80 (96.4%)	87 (79.1%)	<0.001	59 (95.2%)	59 (95.2%)	>0.999
Dyslipidemia	31 (37.3%)	24 (21.8%)	0.018	16 (25.8%)	15 (24.2%)	0.836
Coronary artery disease	34 (41.0%)	22 (20.0%)	0.001	21 (33.9%)	18 (29.0%)	0.562
Heart failure	22 (26.5%)	15 (13.6%)	0.025	14 (22.6%)	11 (17.7%)	0.502
Cerebrovascular disease	31 (37.3%)	24 (21.8%)	0.018	5 (8.1%)	8 (12.9%)	0.379
Parathyroidectomy history	7 (8.4%)	18 (16.4%)	0.104	6 (9.7%)	6 (9.7%)	>0.999
Medications						
ACEI/ARB	29 (34.9%)	24 (21.8%)	0.043	23 (37.1%)	15 (24.2%)	0.119
Glucose lowering drugs	34 (41.0%)	23 (20.9%)	0.003	20 (32.3%)	19 (30.6%)	0.847
Sulfonylurea	14 (16.9%)	13 (11.8%)	0.317	6 (9.7%)	11 (17.7%)	0.192
Dipeptidyl peptidase 4 inhibitors	28 (33.7%)	13 (11.8%)	<0.001	17 (27.4%)	11 (17.7%)	0.198
Insulin	17 (20.5%)	10 (9.1%)	0.024	9 (14.5%)	8 (12.9%)	0.794
Statin	29 (34.9%)	17 (15.5%)	0.002	17 (27.4%)	12 (19.4%)	0.289
Calcium carbonate	67 (80.7%)	94 (85.5%)	0.382	51 (82.3%)	50 (80.6%)	0.817
Proton pump inhibitors	13 (15.7%)	10 (9.1%)	0.163	9 (14.5%)	7 (11.3%)	0.592
Clinical laboratory data						
Hemoglobin (g/dL)	10.62 ± 1.14	10.71 ± 1.41	0.650	10.6 ± 1.05	10.74 ± 1.49	0.555
Albumin (g/dL)	3.52 ± 0.51	3.56 ± 0.46	0.538	3.53 ± 0.46	3.54 ± 0.47	0.902
Total cholesterol (mg/dL)	154.01 ± 33.75	161.89 ± 33.62	0.109	151.94 ± 33.57	163.51 ± 35.30	0.064
Triglyceride (mg/dL)	140.52 ± 103.77	129.61 ± 90.35	0.437	136.21 ± 105.99	131.14 ± 95.51	0.780
High sensitivity CRP (mg/dL)	2.15 ± 4.65	2.5 ± 4.21	0.589	2.45 ± 5.23	2.21 ± 3.95	0.779
Sodium (mmol/L)	136.92 ± 2.68	137.07 ± 2.62	0.700	137.19 ± 2.80	136.64 ± 2.44	0.241
Potassium (mmol/L)	4.73 ± 0.68	4.61 ± 0.62	0.195	4.77 ± 0.66	4.65 ± 0.65	0.294
Total calcium (mg/dL)	9.15 ± 0.86	9.29 ± 0.94	0.277	9.19 ± 0.92	9.25 ± 0.86	0.683
Phosphate (mg/dL)	5.08 ± 1.21	4.95 ± 1.24	0.453	5.16 ± 1.15	5.09 ± 1.35	0.768
Parathyroid hormone (pg/mL)	376.53 ± 338.79	383.5 ± 278.13	0.876	394.16 ± 370.62	357.29 ± 245.84	0.515
Serum iron (μg/dL)	63.57 ± 26.73	65.85 ± 21.16	0.508	63.94 ± 26.61	67.52 ± 22.93	0.424
Ferritin (ng/mL)	567.53 ± 549.64	496.67 ± 377.33	0.291	534.93 ± 330.67	538.54 ± 413.54	0.957
nPCR (g/kg/day)	1.12 ± 0.21	1.16 ± 0.27	0.326	1.12 ± 0.20	1.18 ± 0.28	0.180
Single pool Kt/V	1.67 ± 0.27	1.65 ± 0.27	0.591	1.67 ± 0.28	1.68 ± 0.27	0.817
Dietary intake (serving/day)						
Meat	0.86 ± 0.57	0.82 ± 0.53	0.652	0.86 ± 0.57	0.74 ± 0.52	0.241
Vegetable	2.01 ± 1.09	1.86 ± 1.11	0.265	2.05 ± 1.06	1.91 ± 1.18	0.499
Fruit	0.93 ± 0.72	0.95 ± 0.72	0.583	0.86 ± 0.63	0.89 ± 0.75	0.837
Bristol stool scale	3.94 ± 1.86	3.74 ± 1.76	0.448	4 ± 1.78	3.71 ± 1.67	0.352

Abbreviation: ACEI/ARB, angiotensin-converting enzyme inhibitors/angiotensin-receptor blockers; CRP, C reactive protein; nPCR, normalized protein catabolic rate.

## Data Availability

Raw FASTQ data files of samples from hemodialysis patients have been deposited in the NCBI Sequence Read Archive database under BioProject accession number PRJNA648014.

## Supplementary Materials

The following are available online at https://www.mdpi.com/2075-4426/11/3/198/s1, Figure S1: Rarefaction curves of the number of OTUs versus the sequencing effort per sample in the full cohort. Figure S2: The relative abundance percentage of intestinal microbiota between β-blocker users and nonusers in the full cohort and propensity score matching cohort. (**A**) Phylum level (**B**) Class level (**C**) Order level. Figure S3: Core microbiome analysis in hemodialysis patients with and without β-blocker used. (**A**) SparCC correlation analysis (genus level using 100 SparCC permutations, 0.35 correlation threshold, and 0.05 *p*-value threshold) in all hemodialysis patients with and without β-blocker used (**B**) Relative abundance and sample prevalence of bacterial genus in β-blocker users and nonusers. Figure S4: Enrichment analysis for predictive Kyoto Encyclopedia of Genes and Genomes (KEGG) metabolic modules between β-blocker users and nonusers in full (before propensity score matching) cohort. No significant KEGG enriched pathways were observed (all *p*-value > 0.05). Figure S5: Enrichment analysis for predictive Kyoto Encyclopedia of Genes and Genomes (KEGG) metabolic modules between β-blocker users and nonusers in propensity score-matched cohort. No significant KEGG enriched pathways were observed (all *p*-value > 0.05). Table S1: Summary table of significant genus difference in hemodialysis patients with and without β-blocker treatment in zero-inflated Gaussian fit model.
