# Supplementary material for: Differences in the Microbial Composition of Hemodialysis Patients Treated with and without β-Blockers
Source: J Pers Med. 2021 Mar 12;11(3):198. doi: 10.3390/jpm11030198 (PMC8002078; doi:10.3390/jpm11030198)
Supplement: Supplementary file 1 [file jpm-11-00198-s001.zip › Supplementary Tables and Figures (20210311).docx]

**SUPPLEMENTAL MATERIALS**

**Differences in the microbial composition of hemodialysis patients treated with and without β-blockers**

Yi-Ting Lin ^1,2,3^, Ting-Yun Lin ^4,5^, Szu-Chun Hung ^4,5^, Po-Yu Liu ^6^, Wei-Chun Hung ^7^, Wei-Chung Tsai ^8^, Yi-Chun Tsai ^2,9,10^, Rachel Ann Delicano ^11^, Yun-Shiuan Chuang ^1^, Mei-Chuan Kuo ^2,10,12^, Yi-Wen Chiu ^2,10,12^ and Ping-Hsun Wu ^2,3,12,^*

^1^ Department of Family Medicine, Kaohsiung Medical University Hospital, Kaohsiung 80708, Taiwan; 960254@kmuh.org.tw (Y.-T.L.); kinkipag@gmail.com (Y.-S.C.)

^2^ Faculty of Medicine, College of Medicine, Kaohsiung Medical University, Kaohsiung 80708, Taiwan; lidam65@yahoo.com.tw (Y.-C.T.);mechku@kmu.edu.tw (M.-C.K.); chiuyiwen@kmu.edu.tw (Y.-W.C.); 970392@kmuh.org.tw (P.-H.W.)

^3^ Graduate Institute of Clinical Medicine, College of Medicine, Kaohsiung Medical University, Kaohsiung 80708, Taiwan

^4^ Division of Nephrology, Taipei Tzu Chi Hospital, Buddhist Tzu Chi Medical Foundation, New Taipei City 231, Taiwan; water_h2o_6@hotmail.com (T.-Y.L); szuchun.hung@gmail.com (S.-C.H)

^5^ School of Medicine, Tzu Chi University, Hualien 97071, Taiwan

^6^ Department of Internal Medicine, College of Medicine, National Taiwan University, Taipei 100225, Taiwan; poyu.liu@gmail.com

^7^ Department of Microbiology and Immunology, Kaohsiung Medical University, Kaohsiung 80708, Taiwan; wchung@kmu.edu.tw

^8^ Division of Cardiology, Department of Internal Medicine, Kaohsiung Medical University Hospital, Kaohsiung Medical University, Kaohsiung 80708, Taiwan; k920265@gap.kmu.edu.tw

^9^ Division of General Medicine, Kaohsiung Medical University Hospital, Kaohsiung Medical University, Kaohsiung 80708, Taiwan

^10^ Faculty of Renal Care, College of Medicine, Kaohsiung Medical University, Kaohsiung 80708, Taiwan

^11^ Institute of Surgical Sciences, Uppsala University, 752 36 Uppsala, Sweden; rachel.delicano@surgsci.uu.se

^12^ Division of Nephrology, Department of Internal Medicine, Kaohsiung Medical University Hospital, Kaohsiung Medical University, Kaohsiung 80708, Taiwan

**Corresponding author:**

Ping-Hsun Wu, MD

Division of Nephrology, Department of Internal Medicine, Kaohsiung Medical University Hospital, Kaohsiung Medical University, Kaohsiung, Taiwan

100 Shih-Chuan 1st Road Kaohsiung 807, Taiwan

Telephone number: 886-7-3121101; Fax number: 886-7-3228721

E-mail address: 970392@ kmuh.org.tw

**Figure S1.** Rarefaction curves of the number of OTUs versus the sequencing effort per sample in the full cohort.


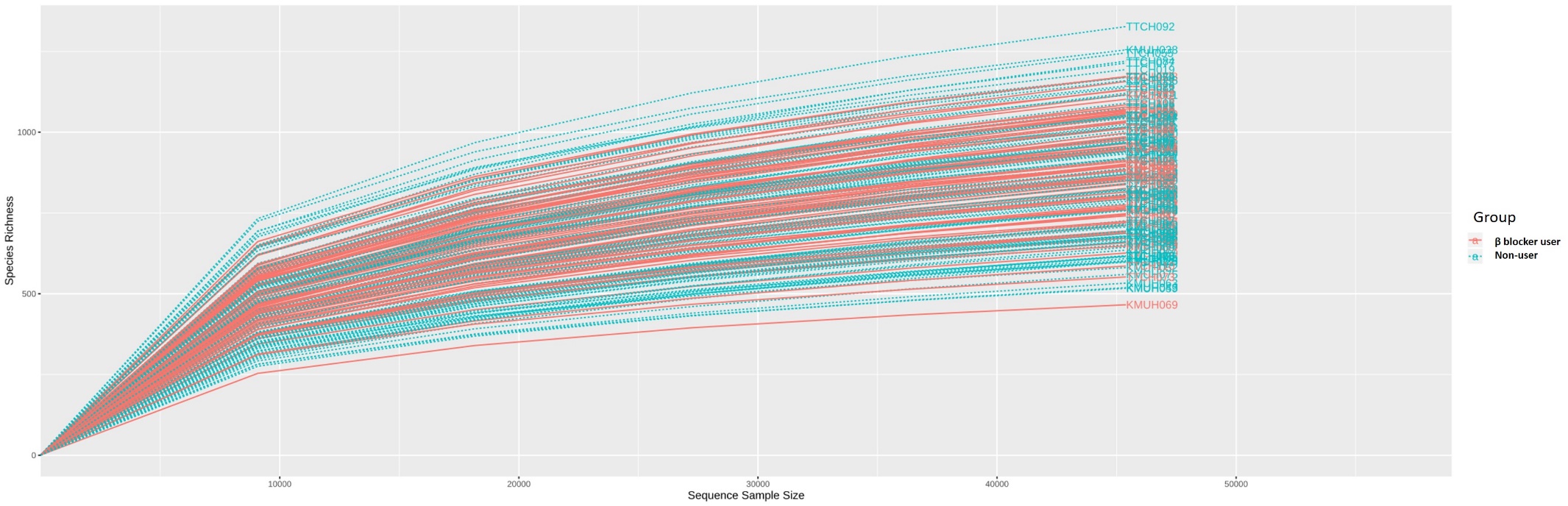


**Figure S2.** The relative abundance percentage of intestinal microbiota between β-blocker users and non-users in the full cohort and propensity score matching cohort. (A) Phylum level (B) Class level (C) Order level.


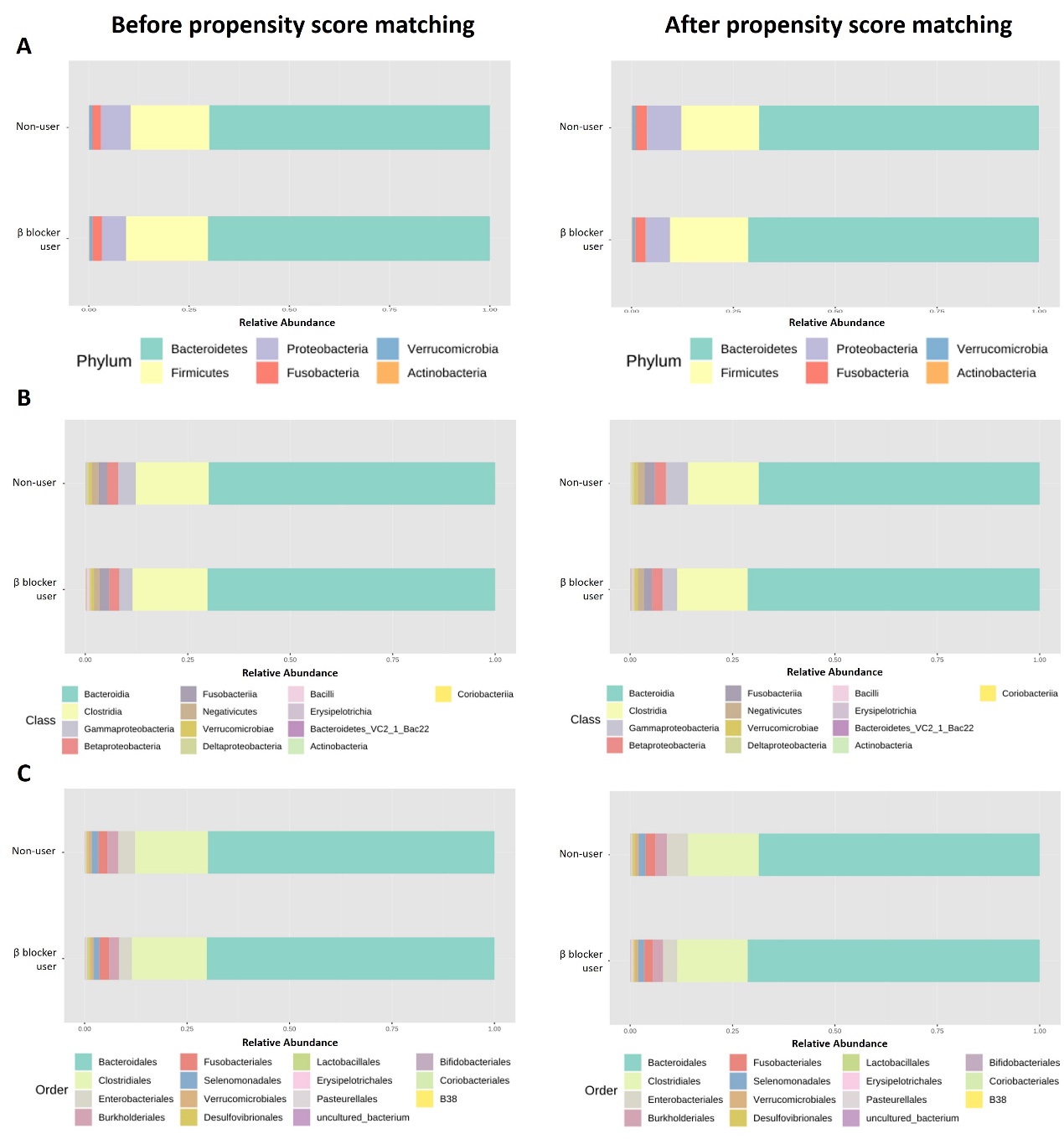


**Figure S3.** Core microbiome analysis in hemodialysis patients with and without β-blocker used. (A) SparCC correlation analysis (genus level using 100 SparCC permutations, 0.35 correlation threshold, and 0.05 *p*-value threshold) in all hemodialysis patients with and without β-blocker used (B) Relative abundance and sample prevalence of bacterial genus in β-blocker users and non-users.


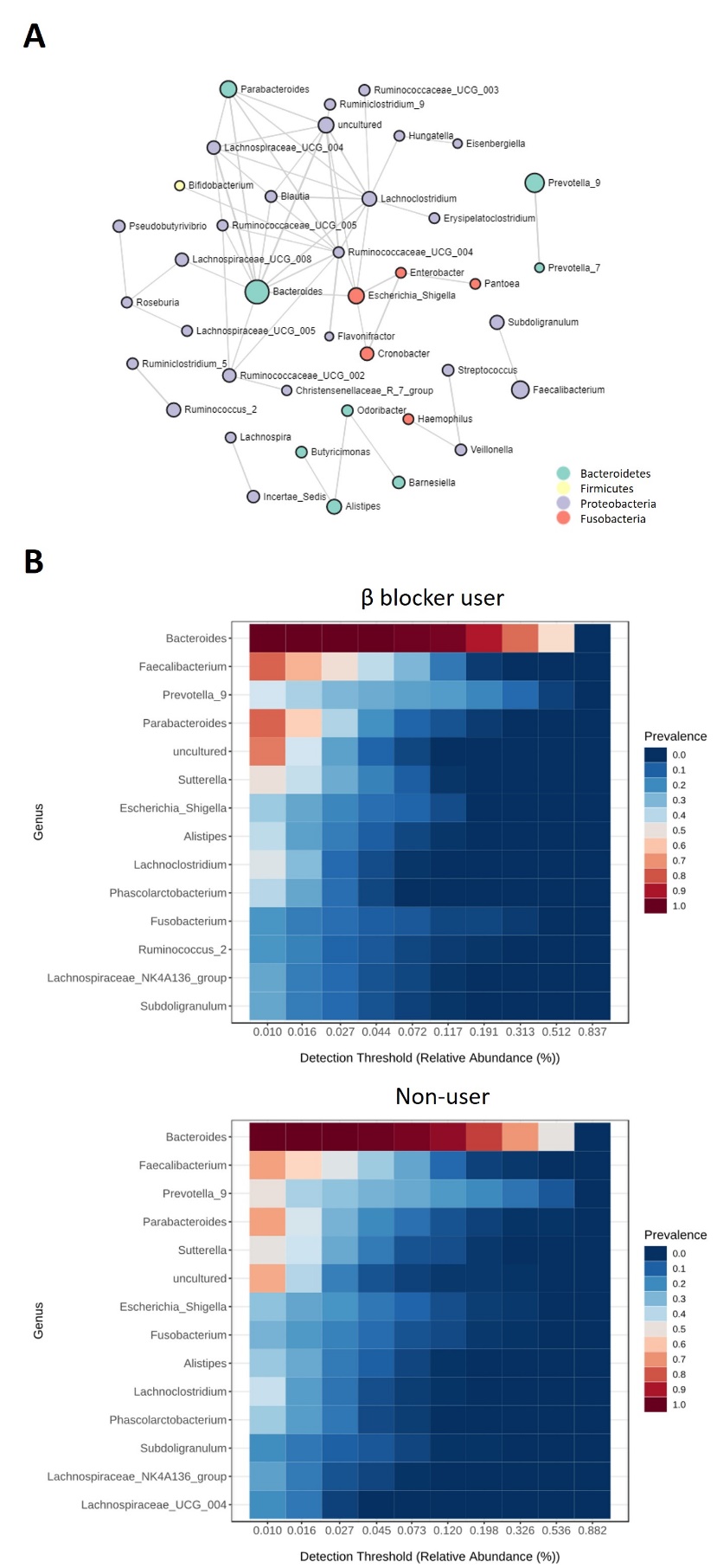


**Figure S4.** Enrichment analysis for predictive Kyoto Encyclopedia of Genes and Genomes (KEGG) metabolic modules between β-blocker users and non-users in full (before propensity score matching) cohort. No significant KEGG enriched pathways were observed (all *p*-value > 0.05).


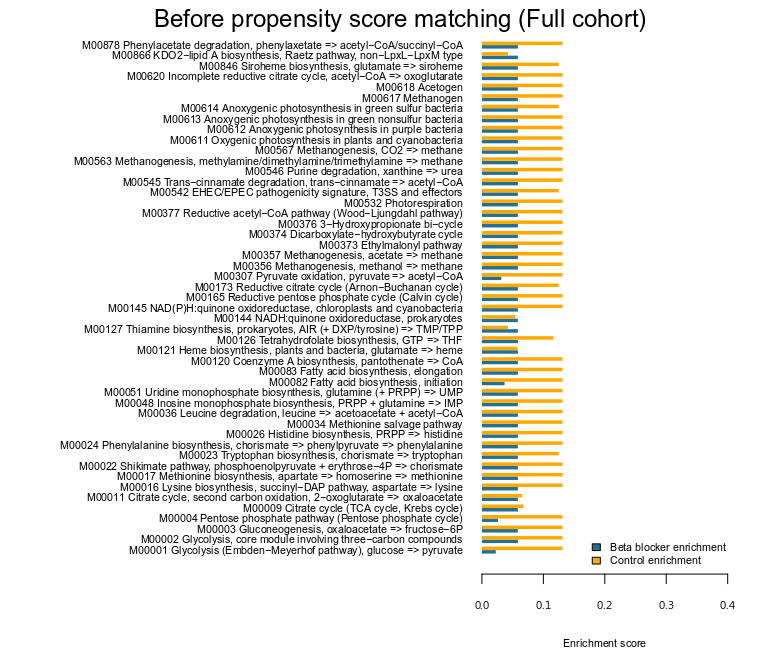


**Figure S5.** Enrichment analysis for predictive Kyoto Encyclopedia of Genes and Genomes (KEGG) metabolic modules between β-blocker users and non-users in propensity score-matched cohort. No significant KEGG enriched pathways were observed (all *p*-value > 0.05).


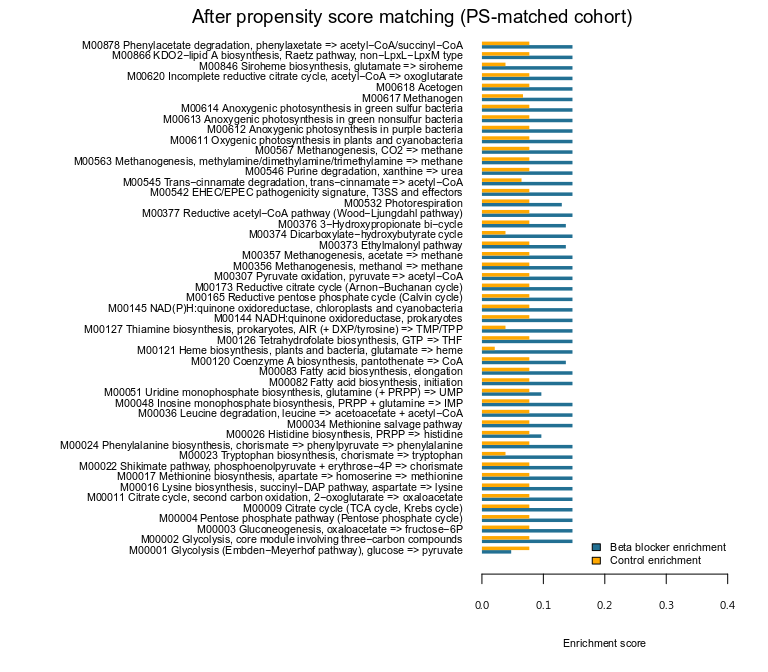


**Table S1.** Summary table of significant genus difference in hemodialysis patients with and without β-blocker treatment in zero-inflated Gaussian fit model

| **Genus** | **Zero-inflated Gaussian fit method** | |
| --- | --- | --- |
|  | **Before propensity score matching** | **After propensity score matching** |
|  | ***p*-value** | ***p*-value** |
| Prevotella_2 | 0.192 | 0.004 |
| **Prevotellaceae NK3B31 group** | **0.030** | **0.005** |
| Subdoligranulum | 0.400 | 0.011 |
| **Flavonifractor** | **0.044** | **0.015** |
| **Tyzzerella** | **0.049** | **0.024** |
| Streptococcus | 0.073 | 0.037 |
| Ruminococcaceae UCG-005 | 0.283 | 0.039 |
| Faecalibacterium | 0.501 | 0.044 |
| Christensenellaceae R7 group | 0.473 | 0.063 |
| Ruminococcaceae UCG-003 | 0.791 | 0.069 |
| Enterobacter | 0.599 | 0.070 |
| Lachnospiraceae UCG-004 | 0.641 | 0.091 |
| Ruminiclostridium 9 | 0.044 | 0.093 |
| Ruminococcaceae UCG-004 | 0.016 | 0.100 |
| Lachnospiraceae UCG-005 | 0.236 | 0.115 |
| Pantoea | 0.711 | 0.140 |
| Anaerotruncus | 0.031 | 0.144 |
| Intestinibacter | 0.496 | 0.148 |
| Eubacterium ventriosum group | 0.488 | 0.187 |
| Erysipelatoclostridium | 0.271 | 0.200 |
| Ruminiclostridium 6 | 0.204 | 0.210 |
| Collinsella | 0.748 | 0.210 |
| Lachnoclostridium | 0.850 | 0.232 |
| Bacteroides | 0.282 | 0.232 |
| Prevotella 9 | 0.796 | 0.244 |
| uncultured | 0.117 | 0.252 |
| Eubacterium coprostanoligenes group | 0.765 | 0.252 |
| Cronobacter | 0.810 | 0.254 |
| Roseburia | 0.303 | 0.274 |
| Lachnospiraceae NC2004 group | 0.573 | 0.311 |
| Ruminiclostridium | 0.314 | 0.312 |
| Eubacterium hallii group | 0.111 | 0.316 |
| Butyricimonas | 0.448 | 0.344 |
| Eisenbergiella | 0.080 | 0.351 |
| Barnesiella | 0.554 | 0.358 |
| Clostridium sensu stricto 1 | 0.778 | 0.375 |
| Lachnospiraceae UCG-010 | 0.778 | 0.382 |
| Phascolarctobacterium | 0.926 | 0.385 |
| Ruminiclostridium 5 | 0.043 | 0.397 |
| Akkermansia | 0.169 | 0.399 |
| Bifidobacterium | 0.651 | 0.422 |
| Haemophilus | 0.534 | 0.444 |
| Lachnospira | 0.613 | 0.446 |
| Fusicatenibacter | 0.887 | 0.460 |
| Sutterella | 0.991 | 0.460 |
| Megamonas | 0.957 | 0.483 |
| Lactonifactor | 0.385 | 0.488 |
| Dorea | 0.897 | 0.551 |
| Desulfovibrio | 0.087 | 0.555 |
| Parabacteroides | 0.194 | 0.573 |
| Pseudobutyrivibrio | 0.910 | 0.577 |
| Odoribacter | 0.761 | 0.577 |
| Intestinimonas | 0.096 | 0.581 |
| Lachnospiraceae NK4A136 group | 0.819 | 0.644 |
| Hungatella | 0.288 | 0.644 |
| Eubacterium ruminantium group | 0.457 | 0.645 |
| Eubacterium oxidoreducens group | 0.651 | 0.658 |
| Bilophila | 0.750 | 0.682 |
| Lachnospiraceae UCG-008 | 0.618 | 0.694 |
| Prevotella 7 | 0.804 | 0.694 |
| Paraprevotella | 0.869 | 0.709 |
| uncultured bacterium | 0.849 | 0.723 |
| Erysipelotrichaceae UCG-003 | 0.739 | 0.822 |
| Escherichia Shigella | 0.915 | 0.838 |
| Parasutterella | 0.326 | 0.858 |
| Fusobacterium | 0.990 | 0.872 |
| Incertae Sedis | 0.959 | 0.874 |
| Veillonella | 0.522 | 0.888 |
| Blautia | 0.829 | 0.894 |
| Ruminococcus 2 | 0.145 | 0.896 |
| Oscillospira | 0.523 | 0.913 |
| Alistipes | 0.650 | 0.921 |
| Ruminococcaceae UCG-013 | 0.745 | 0.925 |
| Ruminococcaceae UCG-002 | 0.388 | 0.928 |
| Anaerostipes | 0.581 | 0.938 |
| Ruminococcus 1 | 0.876 | 0.958 |
| Ruminococcaceae NK4A214 group | 0.498 | 0.979 |
| Family XIII AD3011 group | 0.018 | - |
| Lachnospiraceae ND3007 group | 0.464 | - |
